# Long QT Interval Syndrome and Female Sex—Review and Case Report

**DOI:** 10.3390/reports8010032

**Published:** 2025-03-17

**Authors:** Lana Maričić, Livija Sušić, Damir Mihić, Nikolina Šego

**Affiliations:** 1 Department of Heart and Vascular Diseases, University Hospital Centre Osijek, 31000 Osijek, Croatia; 2 Faculty of Medicine, University J.J. Strossmayer Osijek, 31000 Osijek, Croatia; 3 Health Center of Osijek-Baranja County, 31000 Osijek, Croatia; 4 Faculty of Dental Medicine and Health, University J.J. Strossmayer Osijek, 31000 Osijek, Croatia

**Keywords:** long QT syndrome, female, genotype, pregnancy, therapeutics

## Abstract

**Background and Clinical Significance:** Congenital LQTS is a life-threatening condition, resulting from a mutation of the gene encoding the cardiac ion channels, which results in prolongation of the ventricular action potential. Genetic screening of family members in symptomatic and asymptomatic patients is crucial for the prevention of sudden cardiac death. There are a number of detected mutations of congenital LQTS, of which the three forms LQT1, LQT2, and LQT3 are the best described. In addition to the described ECG morphology, the key triggers and treatment approach are described. This emphasizes even more the importance of timely screening of these patients, and the decision for therapy. It should be emphasized that the phenotypic manifestations significantly depend on the affected genes. The guidelines in the treatment approach are very clear, although it should be emphasized that beta blockers are the first and basic treatment therapy. The therapeutic choice is narrowed especially if they are not effective. **Case Presentation**: This is a case report of a young woman diagnosed with LQTS who was confirmed to have KCNH2 mutations through genetic analysis. The same mutation was also confirmed in her children. Changes in the therapeutic approach are described, and the use of beta blockers, depending on the symptoms and drug tolerance. Especially in the postpartum period, due to reduced progesterone levels, in this case, the patient was implanted with a cardioverter defibrillator. **Conclusions**: It should be emphasized that timely recognition is essential for early diagnosis, regular control, timely initiation of treatment, and prevention of adverse events.

## 1. Introduction and Clinical Significance

Long QT syndrome (LQTS) is an inherited arrhythmia characterized by a prolonged QT interval and T-wave abnormalities on the electrocardiogram (ECG). Clinically, it most commonly presents with episodes of syncope, although the first manifestation may be sudden cardiac death due to torsades de pointes. LQTS is a cardiovascular disorder involving abnormal repolarization of the heart, resulting in a prolonged QT interval and T-wave abnormalities on the ECG [1,2]. The syndrome was first recognized in 1957, when a paper was published describing four children of unrelated parents who were affected by congenital deafness in combination with an uncommon heart disease, which caused syncopal episodes and an ECG finding of a prolonged QT interval [3]. In 1963, Romano et al. described a case of a rare cardiac arrhythmia in a two-year-old child, with syncopal attacks caused by ventricular fibrillation [4]. The following year, Ward reported a new familial cardiac syndrome [5]. Since 1975, the term “long QT syndrome” has become standard, and the syndrome is divided into two main types. Jervell–Lange-Nielsen syndrome is characterized by bilateral sensorineural hearing loss and a QTc interval greater than 500, while Romano–Ward syndrome is a more common form that is inherited in an autosomal dominant manner and does not include congenital deafness [5]. The genetic basis of LQTS was identified in the mid-1990s, and all known genes associated with this disorder encode subunits of cardiac ion channels or proteins involved in the modification of ion currents. Mutations in these genes (*KCNQ1*, *KCNH2*, *KCNE1*, *KCNE2*, *CACNA1c*, *CAV3*, *SCN5A*, *SCN4B*) lead to a prolongation of the action potential duration. The most common form of LQTS (LQT1) is caused by mutations in the *KCNQ1* gene, and about half of patients who have been genotyped have mutations in this gene. So far, 17 subtypes of congenital LQTS have been described, each associated with a different gene. The three most common subtypes include LQT1, which is caused by mutations in the *KCNQ1* (I_Ks_ potassium channel [Kv7.1]) gene; LQT2, which results from mutations in the *KCNH2* (I_Kr_ potassium channel [Kv11.1]) gene; and LQT3, which is associated with mutations in the *SCN5A* gene (I_na_ sodium channel [Na_V_1.5]) [6]. Together, these genetic subtypes account for approximately 65% of all LQTS cases, and approximately 80% of genotype-positive cases [7,8,9].

Pathophysiology

The duration of the QT interval is strongly correlated with the duration of the ventricular action potential, which in turn is determined by the opening and/or closing of ion channels in the heart [10]. Depolarization is caused by the influx of positive ions (such as sodium and calcium), while repolarization involves their efflux. Any disorder at the level of ion channels that causes the accumulation of positive ions within the cell will result in a prolongation of the action potential, and thus the QT interval. A prolonged QT interval can be either acquired or congenital. Congenital prolongation of the QT interval is caused by mutations in the genes encoding ion channel proteins, which leads to their inadequate functioning and the accumulation of intracellular positive ions. In the long QT syndrome, disorders of the late sodium influx in phase 2, slow calcium influx, as well as the rectifier potassium currents in phases 3 and 4 (I_Kr_, I_Ks_, I_K1_), play a key role. Each LQTS genotype has specific triggers for arrhythmia onset and characteristic electrocardiographic features [11,12,13]. Arrhythmias in LQTS typically arise in the latter part of the ventricular action potential, where severe prolongation of the potential duration causes an early after depolarization. This depolarization may reach the threshold for a rapid after sodium current, leading to ventricular arrhythmias that rapidly become polymorphic and may degenerate into serious forms of cardiac arrhythmias.

LQT1

*KCNQ1* encodes the α-subunit of the K_v_ 7.1-volt-gated potassium channel and is responsible for the slow outward potassium current (I_Ks_) during cardiac action potential repolarization. I_Ks_ is an outgoing potassium current that is generated by the *KCNQ1/KCNE1* complex and is one of the potassium currents that contribute to the end state of the cardiac action potential. I_Ks_ mainly contributes to repolarization of the action potential during adrenergic stimulation, when its amplitude increases and accelerates the active process [14,15,16].

LQT2

LQTS type 2 (LQTS2) is caused by mutations in the *KCNH2* gene, leading to a reduction in the fast-acting delayed K+ rectifier current and loss of function of the human ether-à-go-go (hERG)-related channel. KCNH2 encodes two α-subunits of channels (hERG 1a and 1b) that are expressed in cardiac tissue, and both subunits are responsible for encoding the voltage-gated α-subunit of potassium channels [17,18,19]. The I_K_r channel is characterized by slow kinetics of activation and deactivation, associated with rapid inactivation and recovery from inactivation, which partly contributes to determining the prolonged plateau phase typical of the ventricular action potential [20].

The rapid inactivation of the hERG channel means that after the depolarization of the cell membrane, the channel opens, but very quickly switches to the inactivated state. Recovery of hERG channels to the closed state (deactivation) is slow, leading to a large K tail current. This feature of current conduction through the hERG channel plays a key role in the electrical excitability of the heart because it controls the duration of the action potential [21,22,23].

LQT3

Mutations in the *SCN5A* gene have been linked to Long QT Type 3 (LQT3) syndrome, with at least three distinct forms of mutations identified that enhance sodium channel function [24,25]. Cardiac sodium channels are crucial for the excitability of myocardial cells and the effective conduction of electrical impulses throughout the heart. Dysfunction of these sodium channels, arising from mutations in the *SCN5A* gene—which encodes the cardiac (I_na_ sodium channel [Nav1.5])—is associated with a range of inherited cardiac syndromes, including LQT3. Sodium channels are not uniformly distributed within the cardiac conduction system and ventricular walls, which affects their functioning. Most *SCN5A* mutations leading to LQT3 disrupt the normal rapid inactivation process of the sodium current. This dysfunction allows the channels to reopen inappropriately, resulting in persistent (late) sodium currents during the plateau phase of the action potential. Consequently, this leads to delayed repolarization and an extended action potential duration. The presence of early after depolarizations, triggered by the prolonged action potential, can provoke torsades de pointes (TdP), a type of polymorphic ventricular tachycardia that significantly increases the risk of sudden cardiac death [26]. Understanding these mechanisms is essential for recognizing the implications of *SCN5A* mutations in LQT3 and developing targeted therapeutic approaches.

Acquired QT interval

Acquired causes, due to electrolyte disturbances (hypokalemia, hypocalcemia, hypomagnesemia) are much more common causes of prolonged QT intervals. Certain drugs also affect these ion channels and lead to the prolongation of the QT interval. All drugs that cause LQTS act by blocking the outward current of I_Kr_, which is mediated by the potassium channel encoded by the *KCNH2* gene. Medicines that lead to prolongation of the QT interval are antiarrhythmics such as sotalol and amiodarone, antibiotics such as macrolides and fluoroquinolones, and antipsychotics such as haloperidol and olanzapine [10].

### 1.1. Genotype–Phenotype Correlation

As of now, 17 subtypes of congenital LQTS have been identified. The genotype appears to be a robust indicator of risk and can help predict responses to antiadrenergic therapy. The severity of ion channel dysfunction and the specific location of mutations significantly influence both the QT interval and the clinical presentation of the syndrome. In patients with LQT1, cardiac events are primarily triggered by adrenergic stimuli, including both physical and emotional stress. Research indicates that 55% of events in this group occur during physical activity, while 14% occur due to excitement, 21% during sleep or rest, and 10% are triggered by other factors [12,26,27]. Notably, men with LQT1 tend to experience their first event at a younger age compared to women and have a higher risk for exercise-induced events. In contrast, women demonstrate a 3.5-fold greater risk for events that occur during sleep or rest [25]. Genotype–phenotype studies have shown that patients with specific LQT1 mutations exhibit heightened sensitivity to sympathetic stimulation, placing them at increased risk for adverse cardiac events [28,29].

In LQT2, most adverse cardiac events are linked to sudden arousal triggers, particularly auditory stimuli, which account for 43% of events, while only 13% are associated with physical activity [30]. Patients with mutations in the pore region of the KCNH2 gene show a significantly elevated risk of arrhythmia-associated events compared to those without such mutations [31,32].

LQT3 patients often present with additional features, including conduction disorders, atrial arrhythmias, bradycardia, and Brugada syndrome. Studies have demonstrated that individuals with LQT3 are more prone to experiencing adverse cardiac events during sleep [12,26]. Understanding these genotype–phenotype relationships is crucial for improving risk assessment and management strategies for patients with different subtypes of congenital LQTS.

Epilepsy and long QT syndrome

A series of clinical studies have shown that cardiac channelopathies not only cause arrhythmias, but can also affect cerebral function and cause epilepsy. Epilepsy-like episodes are common in patients with a prolonged QT interval. According to research, as many as 34% of patients with LQTS have epilepsy. The genes associated with LQTS, *KCNQ1*, *KCNH2*, and *SCN5A*, are common causes of epilepsy. The Arg 744* variant of *KCNH2* has previously been reported in people with epilepsy or LQTS. The above indicates that the treatment of LQTS should be viewed in the broader context of possible involvement of the brain and heart, not only in terms of the diagnosis of epilepsy but also in the approach to treatment, due to the possible drug effect on the prolongation of the QT interval [33].

Women and long QT interval syndrome

In long QT syndrome, gender is an independent risk factor for the development of malignant arrhythmias, with women at higher risk. The phenotypic dominance of women in LQTS is slight, but the risk of arrhythmias increases after adolescence due to differences in sex hormones. Although women are considered to have a low risk of arrhythmias during pregnancy, this risk increases after childbirth and during menopause and perimenopause [34].

Studies have shown that even in healthy individuals, there are differences in the electrical profile, which are not caused by differences in body weight, left ventricular mass, or height. These differences are not present at birth, but after puberty, women have a longer baseline QTc interval compared to men [35,36]. The change in QTc interval after puberty may be related to changes in sex hormone levels. The mechanisms by which sex hormones affect cardiac repolarization are still not fully understood, but mechanistic studies indicate that sex hormones differentially affect potassium and calcium channels. Progesterone and estrogen, lipophilic gonadal steroid hormones, have long-term effects on transcriptional regulation via nuclear receptors [37,38]. Progesterone has a short-term effect on shortening the duration of the action potential and provides protection against arrhythmias by modulatory action on slowly activating delayed rectifier potassium currents (I_Ks_) and L-type calcium currents (I_a_, L) [39]. Estrogen, on the other hand, has both short-term and genomic effects on the I_Kr_ channel, reducing its expression and prolonging ventricular repolarization [40,41]. Studies have also shown that progesterone and testosterone decrease calcium flux and increase potassium channel flux, resulting in a shorter QTc interval [34,37]. Estradiol may act as a proarrhythmic agent in LQTS2, as it is associated with the interaction of estradiol with certain potassium currents. The effects of progesterone on the QT interval are associated with an increase in intracellular calcium concentration [42]. After menopause, a woman’s hormonal status becomes complex, with almost undetectable levels of progesterone and androgens, while estrogen levels are low but still measurable, especially in women with higher body fat. These physiologic changes, together with hormonal fluctuations during the menopausal transition and the decline in hormones in postmenopause, pose a significant risk for women with LQT2. Low estrogen levels prolong ventricular repolarization by inhibiting I_Kr,_ while progesterone has a protective effect on I_Ks_ and Ia, L. Reduced protective effects of testosterone on I_Kr_ also play a role [43].

Studies have shown that menopause is not associated with an increased risk of cardiac events in women with LQT1 syndrome, because carriers of this genotype have mutations affecting the I_Ks_ channel. Estrogen and decreased testosterone levels during menopause act mainly to prolong the QT interval by inhibiting I_Kr_, while the improvement in I_Kr_ is lost [43].

### 1.2. Pregnancy and Long QT Interval Syndrome

During pregnancy, various gradual physiological changes occur, including cardiac remodeling and an increase in cardiac output. The rise in heart rate associated with pregnancy leads to a shortening of the QT interval, which may offer protective effects for patients with LQTS. Conversely, the postpartum period is marked by rapid hemodynamic shifts and a heightened risk of life-threatening arrhythmias. Research indicates that fluctuations in sex hormone levels contribute to the differing proarrhythmic effects observed at various life stages. Specifically, progesterone exhibits a favorable antiarrhythmic effect, which is particularly beneficial during pregnancy, while estrogen is associated with proarrhythmic tendencies [44].

Additionally, the decline in progesterone levels during the postpartum period is linked to an increased incidence of postpartum arrhythmias and sudden cardiac death (SCD) in patients with LQT2 [45]. Estradiol influences the dispersion of the action potential, leading to modifications that increase the risk of ventricular tachycardia and SCD [46].

A multicentric study conducted in 2022 found that 80% of pregnancies in women with familial LQTS resulted in live births at term via vaginal delivery. However, it was noteworthy that the rates of stillbirths (4%) and miscarriages (16%) among inherited LQTS cases were significantly higher compared to the general population. This discrepancy cannot be solely attributed to fetal ventricular arrhythmias, but is more likely due to alterations in placental and myometrial function in mothers who are LQTS positive [47].

### 1.3. Therapeutic Approach in Long QT Interval Syndrome

The current literature describes three possible treatment principles, beta blockers, left cardiac sympathetic denervation, and implantable cardioverter–defibrillators. Non-selective beta blockers are the mainstay of treatment in long QT interval syndrome. They form the basis of treatment for genetically proven LQTS patients, and those with a normal QTc interval (i.e., “phenotypically concealed LQTS”) [48]. Propranolol and nadolol stand out from this group. Propranolol stands out as the only beta blocker available in liquid form, which is advantageous in the treatment of children, especially in infancy [49]. Atenolol and metoprolol proved to be less effective. All patients, whether asymptomatic or symptomatic, should avoid drugs that cause QT interval prolongation, as well as stimuli, “triggers” that can cause malignant arrhythmias. Therapy should be started with the use of beta blockers. They exert an antiarrhythmic effect by blocking the adrenergic influx of calcium, except propranolol, which reduces the late sodium influx [49]. The efficacy of beta blockers varies, that is, the efficacy depends on the intrinsic properties of each beta blocker, its pharmacodynamics and pharmacokinetics, as well as the individual characteristics of the patient, including their gender, age, and corrected QT interval. Studies have shown that the effectiveness of beta blockers is the highest in people with the LQT1 mutation, and it is less in those with the LQT2 mutation, and they are least effective in those with the LQT3 mutation [50]. Nadolol, a long-acting, hydrophilic, non-selective beta blocker, with a long half-elimination time, is considered most effective in beta blocker therapy because of its pharmacodynamic criteria and membrane stabilization [51]. The second most effective beta blocker in LQT1 treatment is atenolol [52]. Propranolol (non-selective beta blocker) and nadolol are equally effective, while the use of bisoprolol and metoprolol is considered significantly less effective. Esmelol, a cardio-selective ultra-short-acting beta blocker, has its place, particularly in emergency situations, because of its availability for parenteral administration [53]. Another pharmacological therapy includes late sodium current blockers (mexiletine, flecainide, and ranolazine); particularly, LQT3 patients are responsive to this group, and research has also shown a beneficial effect on LQT2 patients [54]. Mexiletine blocks the late sodium current and is a good alternative in cases of beta blocker therapy failure, primarily in LQT3 patients, and less so in LQT2/LQT1 patients [55]. Flecainide—a type IC sodium channel blocker—has proved effective in treating patients with the LQT3 mutation and shortening of the QT interval [56]. Ranolazine represents an alternative therapy to flecainide in patients with LQT3 mutations; although it reduces the peak sodium influx, it does not show the same efficiency [57].

The recommendations for the use of an implantable cardioverter defibrillator (ICD) are defined in the HRS/EHRA/APHRS consensus statement and apply to patients diagnosed with long QT syndrome (LQTS) who have survived cardiac arrest (Class I). The indications also apply to patients who have experienced repeated syncopal episodes despite receiving β-blocker therapy. In addition, patients with a QTc interval longer than 550 ms or with indicators of electrical instability, such as T-wave alternants, are classified as high risk [58]. Left cardiac sympathetic denervation (LCSD) is a surgical procedure that involves resection of the lower half of the stellate ganglion and the left sympathetic chain within the T1 to T4 levels. This procedure leads to a decrease in sympathetic activity and the release of norepinephrine in the heart. LCSD is most often used in extremely high-risk patients, to bridge the treatment gap between pharmacological therapy and the use of an ICD [59]. Video-assisted thoracoscopic left heart sympathetic denervation (VATS-LCSD) is a less invasive approach that provides a viable treatment option for individuals with LQTS who do not respond well to beta blockers [60].

### 1.4. Gene Therapy

Stem cell therapy involves the use of pluripotent stem cells to regenerate or replace dysfunctional cardiac tissue. Allosteric ion channel modulators and gene-specific therapies are being developed to target specific subtypes of LQTS and improve the specificity and efficacy of treatment. An animal model study showed the possibility of therapy effectiveness in LQT1, based on *KCNQ1* gene suppression and its replacement via adeno-associated virus serotype 9, which is applied in vivo in the aortic root area. The results showed effectiveness at reducing action potential as well as QTc interval [61]. The future of therapeutic possibilities certainly derives from the application of induced pluripotent stem cells, which represents a personalized approach to treatment and are derived from modern genome sequencing technologies [62].

The first line of treatment in pregnancy is beta blockers; depending on the response, mexiletine is added, when it is necessary to avoid hypokalemia and hypomagnesemia. In stubborn cases, implantation of a cardioverter defibrillator is required, which is recommended after 8 weeks of pregnancy. Left cardiac sympathetic denervation (LCSD) should be postponed until the postpartum period [63]. Acute treatment of a malignant disorder includes the first-line iv, or, per os, beta blocker application, as well as the second-line administration of MgSO_4_, lidocaine, and mexiletine, and a temporary pacemaker implantation.

## 2. Case Presentation

**Figure 1:**
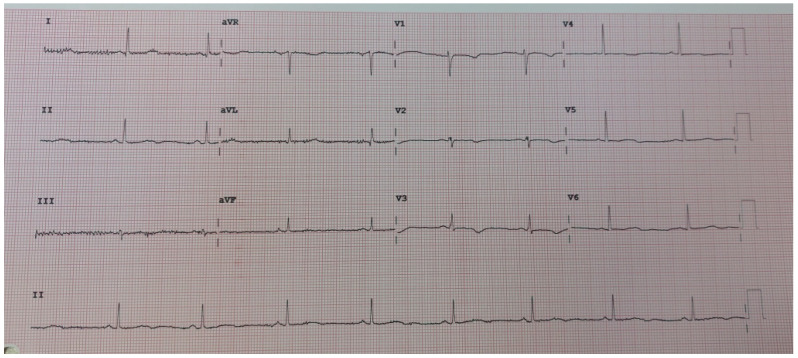
Control ECG after 4 weeks of nadolol therapy, QT interval 500 ms.

Case history: A female patient was diagnosed with a prolonged QT interval 6 years ago, at the age of 35, and then bisoprolol therapy was initiated. Since the age of 12, the patient was managed for epilepsy by a neurologist and had her last grand mal 5 years ago. At the age of 18, she had a pituitary microadenoma surgery. During the first pregnancy, bisoprolol therapy was changed to metoprolol 25 mg, electrocardiographically without prolonged QT interval. There were no neurological deficits, or impaired consciousness. Holter monitoring has been performed multiple times, and there were no cardiac rhythm disorders. The pregnancy was completed by cesarean section at 38 weeks of gestation. In the postpartum period, with metoprolol therapy, VT was detected using Holter monitoring, and the average QT interval duration was 432 ms. Metoprolol was excluded from the therapy, propranolol 3 × 40 mg was introduced, and an ICD was implanted. In the 10-month follow-up period after implantation, no malignant arrhythmias were detected. After the activation, the patient’s second pregnancy was also confirmed. During the second pregnancy, on propranolol 2 × 40 mg therapy, no malignant arrhythmias were detected, and this pregnancy was terminated by cesarean section at 37 weeks of gestation. **Result of genetic test**: Genetic testing of mother and children revealed KCNH2 mutations in different ion channel genes. Half of LQT2 patients remain asymptomatic until the age of 16. Symptoms in LQT2 are triggered by a variety of causes, including emotions in 43% of cases, auditory experiences in 26% of cases, and exercise in 13% of cases. Furthermore, in half of the cases, the symptoms occur during rest or sleep. In the case of this patient, the symptoms occur after a sleepless night, or after the alarm clock activation. The aforementioned is in line with the described symptoms occurring as a part of this syndrome. **Treatment**: In the postpartum period, ventricular fibrillation was detected, as well as ICD activation on two occasions, which led to propranolol dose escalation to 2 × 80 mg; then, due to recurrence, the dose was increased to a maximum of 120 + 120 mg gradually. Since one ICD activation occurred on the maximum dosage, mexiletine was introduced. The patient had no ICD activation for the last 3 months, nor registered rhythm disorders. At the regular check-up, mexiletine was stopped from the therapy because the patient stated that she felt disturbed, but she had no feeling of palpitations. On propranolol therapy, she feels palpitations again, and she had reactivation of the ICD, and detected VF. Propranolol was stopped from the therapy, and nadolol was introduced. **Actual outcome**: After 4 weeks of taking nadolol, there were no palpitations or ICD activation. See Figure 1.

## 3. Discussion

Congenital LQTS is a life-threatening condition, occurring as a result of mutations in the genes encoding cardiac ion channels, resulting in prolongation of the ventricular action potential. Genetic screening of family members of symptomatic and asymptomatic patients is crucial for sudden cardiac death prevention. There is a variety of congenital LQTS mutations; among those, LQT1, LQT2, and LQT3 are best described. In addition to the described ECG morphology, the key triggers and treatment approach are described. This emphasizes even more the importance of timely screening of these patients and therapy decisions. It is important that phenotypic manifestations largely depend on the affected genes. There are clear treatment approach guidelines, and it should be emphasized that beta blockers are the basic and first treatment line. If these are not effective, the therapeutic choice is limited. Considering females, symptom fluctuations depend on the level of hormones in certain periods of the cycle, and also, pregnancy and the postpartum period are unique periods. Precisely, the postpartum period, due to the reduced progesterone level, represents a period suitable for the development of malignant arrhythmias. It should be emphasized that timely detection is essential for early diagnosis, regular control, timely initiation of treatment, and prevention of adverse events.

## 4. Conclusions

Congenital long QT syndrome is characterized by numerous oscillations in its course, and it requires a timely therapeutic approach. In this case report, you can see that certain life periods and hormonal changes required a different therapeutic approach. In addition to effectiveness, side effects that can represent limitations in everyday life play a role in the therapeutic approach.

## Data Availability

The original contributions presented in this study are included in the article. Further inquiries can be directed to the corresponding author.

## Abbreviations

The following abbreviations are used in this manuscript:

LQTS	long QT syndrome
ICD	implantable cardioverter defibrillator
LCSD	left cardiac sympathetic denervation
